# Hydroxychloroquine as an Adjunct Therapy for Diabetes in Pregnancy

**DOI:** 10.3390/ijms25179681

**Published:** 2024-09-06

**Authors:** Nurul Iftida Basri, Padma Murthi, Rahana Abd Rahman

**Affiliations:** 1 Department of Obstetrics and Gynecology, Faculty of Medicine, National University of Malaysia, Kuala Lumpur 56000, Malaysia; 2 Department of Obstetrics and Gynecology, Faculty of Medicine and Health Sciences, Universiti Putra Malaysia, Serdang 43400, Malaysia; 3 Department of Pharmacology, Monash Biomedicine Discovery Institute, Monash University, Clayton, VIC 3800, Australia; padma.murthi@monash.edu

**Keywords:** diabetes, pregnancy, placenta, hydroxychloroquine, inflammation

## Abstract

This review discusses the pathophysiology of diabetes in pregnancy in relation to the placental function. We review the potential use of hydroxychloroquine in improving pregnancy outcomes affected by diabetes. The review focuses on the mechanism of action of hydroxychloroquine and its potential effects on diabetes. There are several pathways in which hydroxychloroquine mediates its effects: through the inflammasome complex, inflammatory cytokines, oxidative stress, modulatory effects, and antihyperglycemic effects. As a safe drug to be used in pregnancy, it is worth exploring the possible use hydroxychloroquine as an adjunct treatment to the current therapy of diabetes in pregnancy.

## 1. Introduction

Diabetes mellitus is a chronic medical condition affecting 529 million people worldwide, with Asia being one of the regions with a higher prevalence [1]. Type 2 diabetes (T2D) occurs as a result of both inadequate insulin secretion due to defective or dysfunctional beta cells of pancreatic islet and insulin resistance when the insulin-sensitive organs fail to respond to insulin effectively. It involves a complex, multifactorial pathophysiology, ranging from the cellular level through to the tissue and organ level. Uncontrolled diabetes subsequently leads to kidney and eye damage, neuropathy, hearing impairment, and increased risk of coronary and cerebral thrombotic disease. Interactions between hereditary, metabolic, and environmental factors play a role in the development of T2D. Evidence from previous studies suggests that modifiable risk factors such as obesity, an unhealthy diet, and a sedentary lifestyle contribute to the onset of T2D [2,3,4].

Gestational diabetes mellitus (GDM), on the other hand, occurs due to failure of normal metabolic adaptations in pregnancy due to the surge of placental hormones, resulting in insulin resistance. As a result, hyperglycemia occurs. The prevalence of GDM has been reported to be between 18 and 37% depending on the studied population [5,6,7]. Risk factors of GDM include obesity, family history, previous history of GDM, and bad obstetric history [4]. Women at risk will be screened for GDM according to their risk factors. Once diagnosed, glucose monitoring is required to ensure good glycemic control. As the pregnancy progresses, women with either GDM or T2D may have enhanced insulin resistance due to the release of cortisol, placenta lactogen, leptin, and placental growth hormone from the placenta [4]. Treatment of both GDM and T2DM are similar including lifestyle changes, dietary modification, metformin, and insulin. Diabetic women may need an increase in medications to maintain normoglycemia as they enter the second half of pregnancy. In most cases, GDM will resolve once the baby is delivered. However, women with GDM are at higher risk of developing T2DM in the future. The risks increased further in women with obesity and with genetic predisposition [4,5].

Obesity is characterized by chronic inflammation of adipose tissue, which is the key player in the development of T2D and GDM [4]. An increase in adipose tissue increases adipocytes, which release excessive free fatty acid and dysregulation of adipokines, resulting in insulin resistance [2,5]. Adipokines play an important role in inflammation. Excess macronutrients in obese individuals stimulate the release of pro-inflammatory cytokines such as tumor necrosis factor-alpha (TNF-α) and interleukin-6 (IL-6), and reduce the production of adiponectin, thereby predisposing one to oxidative stress. [4]. These pro-inflammatory cytokines and oxidative stress are toxic to the beta cells of the pancreas, inducing apoptosis of pancreatic islet cells [8]. This is further enhanced by glucose toxicity, which leads to exacerbated inflammation and beta cell damage [2]. Disruption of the islet cells’ integrity, improper glucagon release, and impaired communication between the cells result in poor insulin regulation and hyperglycemia [8].

In younger populations, there is a higher prevalence of T2D in men as compared to women [9]. The difference is mainly due to the genetics and changes in the sex hormones among women prior to menopause, behavior, lifestyle, and attitude, which influence the risk and progression of diabetes. Research showed that premenopausal women have higher insulin secretion, hepatic insulin sensitivity, and skeletal muscle despite their higher adipose tissue content for a given body mass index (BMI) [9,10]. In contrast, men develop T2D at a lower BMI at a younger age due to the absence of the protective effect of the hormonal influences that are observed in women. Accumulation of the gynoid pattern of fat in premenopausal women further enhances the production of beneficial adipokines, which is negatively associated with T2D and cardiovascular disease [11]. This later leads to lower fasting glucose and HbA1c level [7,10]. However, in the menopausal state, fat distribution changes from a gynoid to an android pattern like in men. An increased testosterone-to-estrogen ratio further heightens the deterioration of glucose tolerance among menopausal women, thus increasing their risk of developing T2D [12].

Pregnancy affected by diabetes has been known to increase the risk of perinatal morbidity and mortality. The risk lies within the placenta, whose function is to provide oxygen and nutrients to the developing fetus while eliminating waste. Diabetes causes several pathological changes such as higher placental weight and volume, villous immaturity, vasculopathy, and fibrinoid necrosis. Placental abnormalities found in pregestational diabetes and gestational diabetes are reported to be similar [13]. The most common findings were defects in villous maturity and increased angiogenesis. In this review, we highlight the pathophysiology of diabetes as a placental disease and the possible mechanism of action of hydroxychloroquine in improving diabetes outcomes.

The first line treatment for diabetes includes lifestyle intervention: education for nutritional therapy and diet, exercise, and weight loss. Several types of lifestyle interventions have been proposed. Group or individual counseling has been utilized in delivering the interventions either by dietitians, diabetic nurses, physicians, trainers, behavioral therapists, or peer counselors [2]. Weight loss has been proven not only to improve glycemic control in diabetic patients, but also to reduce the risk of diabetes among those at risk [14,15]. An increase in physical activity relates to improvement in insulin resistance and weight reduction, thus leading to better glycemic control [16]. Nutritional therapy, on the other hand, aims to promote a healthy eating pattern and appropriate portions to address an individual’s needs while maintaining the pleasure of eating. Diabetic patients are encouraged to increase fiber intake as it may lower hemoglobin A1c (HbA1c) levels [17]. Carbohydrates, proteins, and fat intake should ideally be addressed individually as people responded differently. Some reported a rapid rise followed by a quick fall, while others reported an extended rise and a slow fall [17]. Hence, each individual’s needs differ according to the body’s response. Other potential benefits of diet are that it will lower the triglyceride and low-density lipid (LDL) levels, increase high-density lipid (HDL) levels, induce weight loss, and lower blood pressure.

Current medications used for T2D focus on improving insulin resistance (metformin, thiazolidinediones), increasing insulin secretion (sulphonylurea) and incretins’ effect (dipeptidyl peptidase 4 inhibitor DPP4-i), increasing glucagon-like peptide-1 receptor agonist (GLP1-RA), and reducing glucose absorption (sodium-glucose cotransporter 2 inhibitors SGLT2-I). Few studies have investigated the use of anti-inflammatory drugs on beta cell function of the pancreas, changes in inflammatory markers, and effects on insulin resistance [18,19,20]. Chloroquine (CQ) and hydroxychloroquine (HCQ), a derivative of quinine, was originally introduced as an antimalarial drug after World War II. It is an immunomodulatory drug with anti-inflammatory actions [19,20,21,22]. It has long been recognized for its role in treating autoimmune diseases such as systemic lupus erythematosus (SLE), Sjogren’s syndrome, and rheumatoid arthritis [21,22]. There is emerging evidence to suggest it has a favorable effect on diabetes [19,23,24,25]. 

Although the mechanism is still unclear, data showed that CQ and HCQ equalize pro- and anti-inflammatory markers, reduce the risk of developing diabetes, improve lipid profiles, and provide better diabetic control by reducing the fasting and postprandial blood glucose. Although there is some knowledge on the effect of CQ and HCQ on SLE and other pregnancy complications, such as pre-eclampsia, there is a paucity of data available on the effect of these treatments in pregnancies complicated with T2D and GDM [26,27,28]. Therefore, this review highlights the recent evidence on the mechanism of action of CQ and HCQ and their potential use in pregnancies complicated with T2D and GDM.

## 2. HCQ Mediated Effects on the Pathophysiology of Diabetes Mellitus

### 2.1. Inflammasome Complex

The inflammasome complex comprises pattern recognition receptors (PRRs), the pro-inflammatory caspase-1, and an adaptor protein that mediates the inflammatory response to either endogenous or exogenous pathogens. [26,27]. Aberrant inflammasome signaling has been linked to the development of metabolic diseases such as diabetes. The PRRs of the inflammasomes recognize either pathogen-associated molecular patterns (PAMPs) or danger-associated molecular patterns (DAMPs) such as reactive oxygen species (ROS), fibrinogen, and heat shock proteins [26,28]. The main purpose of PRRs is to promote phagocytosis and activate inflammatory pathways to induce immunity [28]. Toll-like receptors (TLRs) and Nod-like receptors (NLRs) are among the families of PRRs identified. 

Diabetes and obesity are both regarded as low-grade inflammatory conditions. TLRs are innate immune cell receptors. They have been identified to mediate chronic inflammatory conditions as these receptors are found in the pancreatic islet cells. About 10 TLRs are found in humans and each TLR contains an ectodomain, transmembrane domain and, a cytoplasmic toll IL-1 receptor (TIR) domain. Upon recognition of specific ligands, such as PAMPs and DAMPs, TLRs elicit an immune response as a defense mechanism and restore the damaged tissues, thus releasing various inflammatory cytokines and immune modulators [29]. TLR4 expression is upregulated with a high level of glucose and saturated fatty acids, hence generating more inflammatory cytokines. Conversely, TLR2 activates the myeloid differentiation factor 88 (MYD88)-dependent pathway upon recognition of PAMPs and DAMPs [30]. Thus, it plays a role in the pathogenesis of immune-related diseases such as T2D. The interaction between TLR2 and its related ligands has been shown to result in the production of pro-inflammatory cytokine and macrophage activation, which contribute to islet cell inflammation [30].

**Figure 1:**
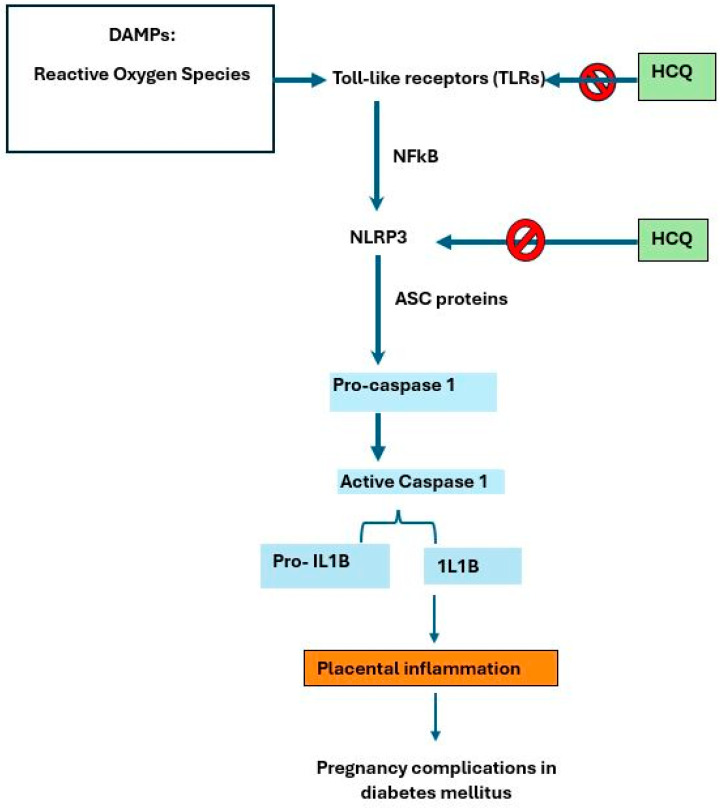
Inflammasomes—mediators of inflammation and diabetes. The activation of the inflammasome complex NLRP3 occurs when DAMPs (such as ROS) interact with Toll-like receptors (TLRs) and activate nuclear factor-kB (NFκβ) [31,32,33,34,35,36]. Caspase-1 activation then ensues, leading to the production of pro-inflammatory cytokines and eventual placental inflammation in diabetes [32,33,34,35]. Targeting the pathway with HCQ may inhibit (red sign showing inhibition) the activation of TLRs or NLRs and mitigate the innate immune signaling which leads to the pathophysiology of diabetes in pregnancy.

The nucleotide-binding oligomeric domain-like receptor containing pyrin domain 3 (NLRP3) inflammasome is an innate immune receptor. It plays a role in metabolism and inflammation, such as in diabetes. In diabetes, its activation causes destructive inflammation to the pancreatic islet cells [31]. An in vivo study found that HCQ inhibits Ca^2+^-activated K^+^ channels, thus impairing the activation of NLRP3 inflammasome complexes [32,33,34,35]. An animal study studying the effect of HCQ on renal injury found that HCQ decreased both pro-inflammatory cytokine production and macrophage and neutrophil infiltration, thus having a downstream effect on NLRP3 inflammasome activation [36]. Apart from inhibiting NLRP3 activator initiation signal, HCQ also prevents the activation of caspase-1 in vitro [32,33,34,35,36]. Another animal study supported the findings that HCQ suppressed the inflammasome activation, suggesting its inhibitory effect on the initiation signal [33]. Other studies found that CQ and HCQ also hinder lysosomal activity and modulate TLRs [35,37]. The expression of NLRP3 was also suppressed in mice affected by nephropathy, which is commonly seen among diabetes patients [35]. Refer to Figure 1.

### 2.2. Inflammatory Cytokines

A high glycemic status induces oxidative stress which then activates inflammation. Excessive adipose tissue in obesity further enhances the inflammation. Several inflammatory markers have been identified as elevated in diabetic patients such as tumor necrosis factor-alpha (TNF-α), C-reactive protein (CRP), interleukin 1βeta (IL-1β), and interleukin-6 (IL-6) [34,38,39]. The increase in pro-inflammatory biomarkers causes dysfunctional pancreatic beta cells, deficient insulin secretion, and action [40,41]. Histologically, abundant cytokine expression and pro-inflammatory macrophage infiltration with fibrosis was seen in the pancreatic islets during the chronic inflammatory process [40,41].

HCQ reduces the synthesis of pro-inflammatory mediators through suppression of the Toll-like receptor (TLR) signaling pathway and inhibits the lysosomal enzyme activity which blocks the antigen presentation [20]. These pathways react to both pathogen- and damage-associated molecular patterns to induce inflammation, thus leading to metabolic syndrome [22]. Various pre-clinical studies showed evidence of its metabolic action on inflammatory mediators. The administration of HCQ to obese arthritic mice lowers insulin resistance, thus lowering the insulin levels and reducing weight. Arthritic mice given HCQ, regardless of whether lean or obese, showed a reduction in IL-1β, TNF-α, and leptin [42]. Messenger ribonucleic acid (mRNA) expression of F4/80, which is a specific marker of macrophages in adipose tissue, was found to be lowered in mice receiving HCQ [42]. Pancreatic islets of diabetic rats exposed to HCQ showed preservation of the beta cells, proliferation and neogenesis of islets of Langerhans, and the absence of inflammatory cells compared to non-HCQ-exposed rats [43].

Experimental animal model studies: Streptozotocin (STZ), a commonly used agent to induce diabetes in animal studies, destroys the beta cells of the pancreas. In STZ-induced diabetic rats treated with 4 weeks of oral HCQ, pro-inflammatory mediators of IL-1β, IL-6, TNF-α, monocyte chemoattractant protein-1 (MCP-1), and transforming growth factor-beta 1 (TGF-B1) dropped significantly [41]. Nine weeks CQ use from 80 mg/week to 250 mg/day reduced TNF-α production, although there was no effect on CRP levels [42]. Nevertheless, a reduction in high-sensitivity CRP levels was seen in another study conducted in India where a combination of HCQ and atorvastatin was used in adults with metabolic syndrome [44].

Randomized controlled trials (RCTs): A randomized trial among insulin-resistant adults prescribed HCQ showed a reduction in IL-6; nonetheless, the authors did not find any reduction in CRP or leptin [38]. An observational study of 250 adults with uncontrolled diabetes on multiple oral hypoglycemic agents (OHAs) showed a reduction in highly sensitive-CRP (hs-CRP) and improvement in glycemic parameters with the addition of HCQ [45]. This was further supported by another trial among patients with uncontrolled T2D on glimepiride and metformin, which also found the level of IL-6, CRP, and adiponectin levels to be significantly improved after the addition of 400 mg of HCQ [21]. In addition, a study among non-diabetic adults with obesity found that HCQ significantly improved adipokine adiponectin levels, which exert anti-inflammatory actions and later improve insulin sensitivity [46]. Hence, the effect of HCQ on reducing the inflammatory markers was promising.

### 2.3. Oxidative Stress and Modulatory Effect

High levels of oxidative stress and radicals play a significant role in the development of metabolic diseases including diabetes. Apoptosis of beta cells due to the activation of reactive oxygen species (ROS) causes impairment of insulin synthesis, resulting in insulin resistance [47]. The rise in ROS levels could be due to the damage and subsequent reduction in antioxidants such as catalase (CAT), superoxide dismutase (SOD) and glutathione peroxidase (GSH-Px) [48]. ROS also react with proteins to produce glycation and oxidative degeneration. This is later indicated by measuring glycated hemoglobin (HbA1c) and fructosamine. Malondialdehyde (MDA) is another biomarker of oxidative stress, and it reacts with proteins and lipids leading to cellular injury [49].

Current oral hypoglycemic agents appear to have hypoglycemic effects without counteracting the development of ROS-mediated organ damage caused by diabetes. Endothelial dysfunction in diabetes occurs due to a decrease in nitric oxide (NO) production and an increase in ROS [24,50]. HCQ, on the other hand, inhibits ROS production and improves NO levels while improving endothelial dysfunction associated with diseases such as diabetes and hypertension [50]. HCQ also disrupts the polymorphonuclear cell function at the therapeutic level [24,51]. An in vitro study showed that HCQ reduced ROS production through sigma-1 receptors [51]. At a higher concentration of HCQ, it may engulf and destroy these cells, acting as an antioxidant. An animal study by Zannah et al. [52] showed that the level of antioxidant activities, SOD and CAT, was significantly low in diabetic rats compared to their normal counterparts [50]. An addition of HCQ to glibenclamide and metformin treatment significantly increased the SOD levels by more than 50% compared to those produced when either of the drugs was used alone [52]. A similar finding was found in the CAT activity, whereby four weeks’ addition of HCQ to either glibenclamide or metformin resulted in enhanced CAT activity [52].

### 2.4. Hyperglycemia

Hyperglycemia leads to several pathologies, in particular biochemical abnormalities and abnormal oxidative status. Enhanced production of free radicals such as ROS and reactive nitrogen species (RNS) and compromised antioxidant activities have been identified in diabetes. A ROS increase also affects the integrity of the cell function, enzymes, and lipid membranes [52]. MDA concentration was found to be significantly higher in diabetic patients, especially in those at risk of cardiovascular disease [53,54]. An in vitro study using human umbilical vein endothelial cells (HUVECs) exposed to 5 and 30 mM glucose contents showed a significant decrease in ROS, nitric oxide (NO), and MDA in all groups treated with HCQ [53]. An addition of HCQ reduces the MDA levels to their initial range in both groups exposed to 5 mM or 30 mM glucose concentration [53].

Studies using in vivo models found that blood glucose levels among alloxan-induced diabetes in rats were significantly reduced following treatment with either a combination of HCQ and metformin or HCQ and glibenclamide [45]. This, however, was not seen in the group treated with HCQ alone [47]. This finding was further supported by another animal model study that compared different disease-modifying anti-rheumatic drugs (DMARDs) and their effects on insulin resistance. In comparison with Leflunomide and Methotrexate, HCQ has a favorable effect on decreasing insulin levels, thereby reducing insulin resistance [55]. Emami et al. [56] revealed that diabetes-induced rats supplemented with HCQ lowered their blood glucose levels while elevating their serum insulin in a dose-dependent manner [57]. Nevertheless, there was no significant effect of fasting blood glucose when oral HCQ was given to healthy rats [54]. The authors postulated that HCQ had minimal effect in the healthy population when compared to the diabetic population.

Evidence from human studies also supports the use of HCQ as a treatment and an adjunct to current diabetes treatments. An observational study among rheumatic disease patients showed a significant reduction in HbA1c levels among those on HCQ compared to those on methotrexate (MTX) [58]. A meta-analysis found that HCQ is effective in lowering plasma glucose level and glycated hemoglobin (HbA1c) levels in addition to reducing the insulin requirement [59]. Another study found similar findings whereby HCQ lowers fasting and postprandial plasma glucose and HbA1c, but there was no effect on insulin levels [56]. A clinical trial was conducted involving 25 patients with metabolic syndrome who were treated with a placebo and different doses of HCQ with a washout period between each phase. The outcomes showed an increase in hepatic insulin sensitivity and a reduction in hepatic gluconeogenesis and serum fasting glucose with an increasing dose. However, at a lower dose of HCQ, there was no effect found on serum fasting glucose, homeostatic model assessment for insulin resistance (HOMA-IR), and insulin sensitivity index (ISI) even when given the long term [60].

## 3. Potential Use of HCQ in Pregnancy

Pregnancy is a physiological low-grade inflammatory state where there is a disturbance of pro- and anti-inflammatory markers [61]. These markers or cytokines are excreted by various cells such as skeletal muscle cells, adipocyte cells, lymphocytes, and natural killer cells. Apart from the secretion of adipokines and cytokines from white adipose tissue, the placenta also acts as an endocrine organ secreting similar cytokines, such as IL-6 and TNF-α [62,63]. Studies have shown that an imbalance of cytokines plays an important role in contributing to insulin resistance in pregnancy [64]. A reduction in insulin sensitivity is more pronounced from 20 weeks gestation onwards [62,63]. Kirwan et al. demonstrated an increase in TNF-α levels during late pregnancy among diabetic pregnant women as compared to normoglycemic pregnant women [65]. TNF-α, however, showed a downward trend in early pregnancy for both diabetic and normoglycemic pregnant women. This may explain the need for higher insulin doses towards the second half of pregnancy. Poorly controlled diabetes is associated with poor maternal and neonatal outcomes, namely preterm birth, stillbirth, fetal macrosomia, and polyhydramnios [65,66,67]. Therefore, the need for a normoglycemic state and low inflammatory status is imperative throughout the pregnancy in determining good clinical outcomes.

Inflammasome complexes which trigger inflammatory responses against metabolic disturbances are important to maintain pregnancy to term. Expression of NLRP1-4 and caspase 1-4 has been reported in placental trophoblasts, the myometrium, and the amniotic membrane at term [7,26]. Disturbances in inflammasome activation have been described in diseases associated with placental inflammation such as pre-eclampsia, gestational diabetes mellitus (GDM), and fetal growth restriction [7,68,69]. GDM shares similar pathophysiology to T2D. Women with T2D genes are at a higher risk of developing GDM during pregnancy. Activation of inflammasome pathways within the low-grade inflammation state of pregnancy results in the production of pro-inflammatory cytokines. This leads to endothelial dysfunction of the placenta and worsens the insulin resistance in GDM [69].

Formation of placental villi begins as early as two weeks post conception. The cytotrophoblasts grow into syncytiotrophoblasts, forming primary chorionic villi [70,71]. The splitting of the extraembryonic mesoderm forms the secondary chorionic villi. The formation of blood cells and vessels gives rise to the tertiary chorionic villi. As the placenta matures, these villi further branch into smaller terminal villi and more peripheral capillaries [70,72]. In GDM, there are defects in these villi formations, resulting in a reduced number and total surface of terminal villi, centrally located capillaries, and thickened basement membranes [70]. Villous edema, which is associated with reduced placental function, is another common abnormality found in GDM [72,73]. A study reported that inflammasome activation in syncytiotrophoblasts induced inflammation by elevation of pro-inflammatory cytokines [26,74]. Similar to pre-eclampsia, placental vascular lesions were also seen in GDM [73]. Fetal thrombotic vasculopathy, fibrinoid necrosis, and intervillous thrombi are common histologic findings as compared to uncomplicated pregnancy [73,74,75].

A hyperglycemic state in pregnancy initiates oxidative stress when there is excessive ROS production and reduction in antioxidants. Sources of ROS include nitric oxide synthase (NOS), NADPH oxidase, and xanthine oxidase [76,77]. As ROS increases, it alters insulin secretion from pancreatic beta cells. This later leads to pancreatic beta cells’ apoptosis and death. This was further accelerated because beta cells have low antioxidant protection. ROS and tissue hypoxia promote pathological angiogenesis, resulting in abnormal vasculature of the placenta [69]. Repeated hypoxia and reoxygenation states lead to abnormal smooth muscle contraction and relaxation of placental vasculature, interrupting the cell morphology and placental barrier function [69]. The imbalance between pro- and antioxidant properties causes alteration in syncytiotrophoblast function. This further results in cellular stress and injury, thus activating the inflammasome [26]. Increased activity of NADPH oxidase (NOX), xanthine oxidase (XO), and reduced catalase (CAT) activity was found in placenta samples from diabetic mothers [74,78,79,80]. With such alterations, a drug that could alter this imbalance of oxidative stress will be beneficial. HCQ is one of the drugs found to inhibit NOX activity on the placenta, thus reducing free radicals’ production [28,55]. In pregnancy, metformin and insulin have been the sole treatment used for diabetic women with proven safety and efficacy. Nevertheless, obstetricians frequently encounter women with inadequate diabetic control despite both metformin and multiple high dosages of insulin. This is then reflected in fetal and maternal complications. The newer hypoglycemic agents lack safety data in pregnancy and pose financial challenges. Hence, the need for an affordable second-line oral hypoglycemic agent in pregnancy is indispensable. In low-resource countries such as India, HCQ has been approved as a third-line treatment for uncontrolled T2D in adults [81,82]. There is growing evidence to support its use in regulating glucose homeostasis in T2D and its safety in pregnancy [78,80,81,82].

HCQ has been safely used for autoimmune and rheumatic diseases in pregnancy. It is known to cross the fetal placental barrier and has proven to improve fetal outcomes in anti-Ro/La-positive mothers [77,81,83]. Chambers et al. performed a prospective study including 500 pregnant women who demonstrated no increased risk of structural birth defects [84]. This finding was further supported by several studies that demonstrated no increased risk of fetal malformations and low birthweight [24,83,84,85]. In addition, HCQ was also found to have protective effects such as decreasing lupus activity, reducing preterm loss, and possibly reducing the risk of pre-eclampsia [33,77,81,85]. A follow-up study also reported that there were no immune effects in infants exposed to HCQ in utero [86,87]. Additionally, evidence showed that HCQ has no detrimental effect on the neurodevelopmental outcomes among offspring exposed to HCQ in pregnancy and breastfeeding [87,88,89,90,91].

The recommended dose for HCQ use is no more than 5 mg/kg/day to prevent the risk of retinopathy [92,93,94]. As HCQ is commercialized as a 200 mg tablet, most patients will receive a daily dose of between 200 and 400 mg of HCQ in cases of lupus and other rheumatic diseases. Lowering the dose by half is required for patients with renal impairment [93]. Based on the current recommendation, we suggest using the same dose limit for the purpose of adjunct treatment of diabetes in achieving normoglycemia in pregnancy, while reducing the risk of retinopathy.

## 4. Conclusions

Over the last 20 years, studies have shown that HCQ can potentially act as an insulin sensitizer, act as an anti-inflammatory agent, and promote insulin secretion in diabetic patients. HCQ exerts its antioxidant effect not only on pancreatic islet cells, but also on the endothelium and the placenta. Multiple studies have proven the safety of HCQ in both pregnancies at risk of developing diabetes and in indicated pregnant populations. Due to its numerous favorable effects and excellent safety profile, further research to look into its application among diabetic patients in pregnancy is worth exploring.

## Data Availability

Not applicable.
